# Supplementary material for: Comparability of the Effectiveness of Different Types of Exercise in the Treatment of Achilles Tendinopathy: A Systematic Review
Source: Healthcare (Basel). 2023 Aug 11;11(16):2268. doi: 10.3390/healthcare11162268 (PMC10454459; doi:10.3390/healthcare11162268)
Supplement: Supplementary file 1 [file healthcare-11-02268-s001.zip › healthcare-2471962-supplementary.pdf]

Table S1. Database search strategy

**PubMed and Scopus**
((("Tendinopathy"[Mesh]) OR "Achilles Tendon"[Mesh] OR "achilles tendinopathy" [tw] OR "achilles tendinitis" [tw] OR "achilles tendon injur*" [tw]) AND ("exercise Therapy"[Mesh] OR "Exercise"[Mesh] OR "Pilates"[Mesh] OR "Clinical Pilates"[Mesh] OR exercis*[tw] OR load*[tw] OR rehab* [tw])) AND ("Treatment Outcome"[Mesh] OR effectiv* [tw]) Filters: Clinical Trial, Randomized Controlled Trial
**Science Direct**
• (tendinopathy OR "Achilles Tendon" OR "achilles tendinopathy") AND ("exercise Therapy" OR Exercise OR exercising OR pilates OR "clinical pilates") AND ("Treatment Outcome" OR effective)<br>• ("achilles tendinitis" OR "achilles tendon injury") AND (load OR loading OR rehabilitation) AND (effectiveness)
**Pedro**
• Achilles tend* exercis* "treatment outcome"<br>• Achilles tend* exercis* effectiv*<br>• Achilles tend* pilates "treatment outcome"<br>• Achilles tend* pilateseffectiv*<br>• Achilles tend* load* "treatment outcome"<br>• Achilles tend* load* effectiv*<br>• Achilles tend* rehab* "treatment outcome"<br>• Achilles tend* rehab* effectiv*

Table S2. Criteria Furlan, et al. (2009) to assess risk of bias in randomized controlled trials

A. 1. Was the method of randomization adequate?	Yes / No / Unclear
B. 2. Was the treatment intervention allocation concealed?	Yes / No / Unclear
C. Was treatment concealment maintained during delivery of the interventions?	Yes / No / Unclear
3. Was there blinding of the therapist during delivery of the intervention?	Yes / No / Unclear
4. Was the patient blinded during the delivery of the intervention?	Yes / No / Unclear
5. Was the outcome assessor blinded to the intervention?	Yes / No / Unclear
D. Are missing outcome data adequately accounted for?	Yes / No / Unclear
6. Is the dropout rate described and acceptable?	Yes / No / Unclear
7. Were all participants analyzed in the group to	Yes / No / Unclear

which they were allocated?	
8. Is the study report free of selective information about the results?	Yes / No / Unclear
E. Other sources of potential bias	Yes / No / Unclear
9. Were the groups similar at baseline on the most important prognostic indicators?	Yes / No / Unclear
10. Were escorts or similar interventions avoided?	Yes / No / Unclear
11. Was compliance acceptable for all groups?	Yes / No / Unclear
12. Was the timing of outcome assessment similar for all groups?	Yes / No / Unclear

Source: Furlan, et al ., (2009)

Table S3. Level of research evidence rating scale

Strong research evidence	Consistent positive (significant) findings from two or more high-quality randomized controlled trials
Moderate research evidence	Consistent positive (significant) findings from multiple low-quality studies and/or clinical trials or one high-quality randomized trial
Limited research evidence	Positive (significant) findings from a low-quality randomized controlled trial or clinical trial
Conflicting research evidence	Conflicting (significant) findings between multiple studies (<75% of studies report consistent findings)
No research evidence was found	Randomized controlled studies available, but no (significant) differences<br>between intervention and control groups
No research evidence	No randomized controlled trials were found

Source : Van Tulder, et al (2003)

Table S4. Characteristics of interventions in the intervention group

Study	Type of exercise	Duration of intervention	Execution of exercises / week	Implementation/ day
Habets et al., 2021	Eccentric Exercise (Alfredson Protocol)	12 weeks	7	2
	Concentric – Eccentric Exercise (SilbernagelProtocol)			1
Beyer et al., 2015	Eccentric exercise	12 weeks	7	2
	High resistance exercises performed at a slow pace		3	-
Yu et al., 2012	Eccentric strenghtening (Combination of Curvin&Stanishand Alfredson protocols)	8 weeks	7	2
	Concentric Strengthening (Mafi Protocol and Stretching)			1
Mafi et al., 2001	Eccentric exercise	12 weeks	7	2
	Concentric exercise			
Niessen-Vertommen et al., 1992	Eccentric exercise (Curvin&Stanish protocol)	12 weeks	6	1
	Concentric exercise			

Table S5. Results and statistical comparisons for outcome measures in the intervention group of studies in the systematic review

Study	Outcome Measure	Baseline M.V (S.D)	Completion of intervention M.V (S.D)	1st period _ M.V (S.D)	2nd period _ M.V (S.D)	p-value baseline - finish	p - value baseline- 1st FOLLOW-UP	p - value baseline- 2nd FOLLOW-UP
Habets et al., 2021	VISA-A	60.7 ± 17.1	-	-	89.4 ± 13.0	-	-	P < 0.001
	VAS-ADL	28.6 ± 22.1	-	-	5.8 ± 8.3	-	-	P = 0.004
	VAS – sports	44.8 ± 26.8	-	-	13.1 ± 20.2	-	-	P = 0.027
	EQ-5D	82.6 ± 8.7	-	-	81.0 ± 20.3	-	-	-
Beyer et al., 2015	VISA-A	58 ± 3.9	72 ± 3.7	84 ± 3.5	-	P < 0.0001	P < 0.0001	-
	VAS (heel)	19 ± 5.0	12 ± 3.6	6 ± 2.6	-	P < 0.0001	P < 0.0001	-
	VAS (running)	49 ± 5.5	20 ± 5.7	12 ± 4.2	-	P < 0.0001	P < 0.0001	-
	A-P, mm	8.3 ± 0.3	8.1 ± 0.4	7.3 ±0.3	-	P < 0.0001	P < 0.0001	-
	Doppler, %	2.8 ± 0.6	2.8 ± 0.5	1.6 ± 0.5	-	P < 0.005	P < 0.005	-
Yu et al., 2012	VAS	5.72 ± 0.89	2.16 ± 0.42	-	-	P < 0.05	-	-
	PTKE, Nm	174.78 ± 9.32	212.26 ±0.02	-	-	P < 0.05	-	-
	PTKF, Nm	118.43 ± 9.88	128.92 ± 5.07	-	-	P < 0.05	-	-
	PTAP, Nm	56.91 ±7.26	66.38± 11.80	-	-	P < 0.05	-	-
	PTAD, Nm	36.97 ± 6.89	44.66 ± 10.18	-	-	P < 0.05	-	-
	BAG, Nm	127.01 ± 4.71	131.41 ±2.16	-	-	P < 0.05	-	-
	EKF, Nm	94.16 ± 15.41	98.07 ± 13.44	-	-	P < 0.05	-	-
	EAP, Nm	48.92 ± 4.42	62.48 ± 9.48	-	-	P < 0.05	-	-
	EAD, Nm	24.75 ± 5.37	41.98 ±3.68	-	-	P < 0.05	-	-
	Total balance index	36.38± 8.51	8.00 ± 5.39	-	-	P < 0.05	-	-
	AP balance index	37.88 ± 4.38	7.38 ± 7.21	-	-	P < 0.05	-	-
	ML balance index	29.38± 4.34	8.25 ± 7.00	-	-	P < 0.05	-	-
	Dexterity	27.75 ± 5.57	51.63 ± 6.78	-	-	P < 0.05	-	-
	Agility	53.13 ± 4.32	68.00 ± 3.46	-	-	P < 0.05	-	-
Mafi et al ., 2001	VAS	69	12	-	-	-	-	-
Niessen-Vertommen et al	Pain	6.0	1.3	-	-	P < 0.01	-	-

Abbreviations: MV, Mean Value; S.D., Standard Deviation; FU, Long-term follow-up; VISA-A, Victorian Institute of Sports Assessment–Achilles; VAS, visual analog scale; ADL, activities of daily living; AP, anterior-posterior, PTKE, peak torque of knee extension; PTKF, peak torque of knee flexion; PTAP, peak torque of ankle plantar flexion; PTAD, peak torque of ankle, dorsiflexion; EKE, endurance of knee extension; EKF, endurance of knee flexion; EAP, endurance of ankle plantar flexion; EAD, endurance of ankle dorsiflexion.

Table S6. Results and statistical comparisons for outcome measures in the control group of systematic review studies

Study	Outcome Measure	Baseline M.V (S.D)	Completion of intervention M.V (S.D)	1st period – Follow-up M.V (S.D)	2nd period – Follow-up M.V (S.D)	p-value start-finish	p - value start - 1st MP	p - value start- 2nd MP
Habets et al., 2021	VISA-A	59.8 ± 22.2	-	-	83.2 ± 22.4	-	-	P < 0.001
	VAS-ADL	28.6 ± 31.8	-	-	9.0 ± 23.0	-	-	P = 0.004
	VAS – sports	46.6 ± 32.6	-	-	12.8 ± 24.6	-	-	P = 0.027
	EQ-5D	77.2 ± 13.1	-	-	77.9 ± 23.4	-	-	-
Beyer et al., 2015	VISA-A	54 ± 3.2	76 ± 3.7	89 ± 2.8	-	P < 0.0001	P < 0.0001	-
	VAS (heel)	29 ± 5.5	7 ± 2.4	5 ± 2.5	-	P < 0.0001	P < 0.0001	-
	VAS (running)	54 ± 5.4	17 ± 4.1	5 ± 2.6	-	P < 0.0001	P < 0.0001	-
	A-P, mm	8.6 ± 0.5	7.9 ± 0.4	6.9 ± 0.3	-	P < 0.0001	P < 0.0001	-
	Doppler, %	4.0 ± 0.8	2.0 ± 0.5	1.0 ± 0.4	-	P < 0.005	P < 0.005	-
Yu et al., 2012	VAS	5.72 ± 0.79	3.26 ± 0.78	-	-	P < 0.05	-	-
	PTKE, Nm	157.92 ± 35.86	188.27 ± 34.36	-	-	P < 0.05	-	-
	PTKF, Nm	114.78 ± 24.75	122.47 ± 19.63	-	-	P < 0.05	-	-
	PTAP, Nm	63.21 ± 6.33	71.18 ± 10.33	-	-	P < 0.05	-	-
	PTAD, Nm	36.73 ± 11.73	43.85 ± 12.16	-	-	P < 0.05	-	-
	BAG, Nm	119.23 ± 26.26	118.46 ± 13.83	-	-	P < 0.05	-	-
	EKF, Nm	91.71 ± 12.50	92.32 ± 20.63	-	-	P < 0.05	-	-
	EAP, Nm	52.91 ± 6.16	59.41 ± 8.36	-	-	P < 0.05	-	-
	EAD, Nm	24.32 ± 4.24	33.02 ± 7.22	-	-	P < 0.05	-	-
	Total balance index	29.00 ± 16.02	22.50 ± 7.52	-	-	P < 0.05	-	-
	AP balance index	40.38 ± 22.74	30.38 ± 21.24	-	-	P < 0.05	-	-
	ML balance index	37.25 ± 11.38	25.00 ± 12.96	-	-	P < 0.05	-	-
	Dexterity	28.75 ± 7.30	49.13 ± 5.41	-	-	P < 0.05	-	-
	Agility	54.63 ± 4.65	64.38 ± 4.47	-	-	P < 0.05	-	-
Mafi et al ., 2001	VAS	63	9	-	-	-	-	-
Niessen-Vertommen et al	Pain	6.5	3.5	-	-	P < 0.01	-	-

Abbreviations: MV,Mean Value; S.D., Standard Deviation; FU, Long-term follow-up; VISA-A, Victorian Institute of Sports Assessment–Achilles; VAS, visual analog scale; ADL, activities of daily living; AP, anterior-posterior, PTKE, peak torque of knee extension; PTKF, peak torque of knee flexion; PTAP, peak torque of ankle plantar flexion; PTAD, peak torque of ankle, dorsiflexion; EKE, endurance of knee extension; EKF, endurance of knee flexion; EAP, endurance of ankle plantar flexion; EAD, endurance of ankle dorsiflexion.

Table S7. Comparison of outcome measures for the intervention and control groups of the Habets study et al ., (2021)

Measuring tool	Baseline– Finish D [CI]	Baseline - 1st period D [CI]	Baseline – 2nd period D [CI]	p-value Baseline – Finish	p - value Baseline – 1st period	p - value Baseline – 2nd period
VISA-A	0.9 [–11.9 ,13.8]	1.1 [–11.8, 14.0]	4.3 [–8.0 ,16.6]	p = 0.885	p = 0 .867	p = 0.479
VAS-ADL	–0.9 [–12.4 ,10.6]	4.5 [–8.1, 17.0]	–0.1 [–10.3, 10.1]	p =0.874	p =0.475	p =0.986
VAS – sports	–0.7 [–18.3, 16.9]	–1.0 [–17.6, 15.7]	2.9 [–12.3, 18.1]	p = 0.936	p = 0.908	p = 0.702
EQ-5D	-	-	-	-	-	-
Abbreviations: D, mean difference between the two groups at this time point; CI, Confidence Interval,VISA-A, Victorian Institute of Sports Assessment–Achilles; VAS, visual analog scale; ADL, activities of daily living						

Table S8. Comparison of outcome measures for the intervention and control groups in the Beyer et al., (2015) and Yu et al., (2012) studies

Study	Outcome Measure	Baseline – Finish		Baseline– 1st period		p-value Baseline– Finish	p - value Baseline– 1st period
		EG D [CI]	CG D [CI]	EG D [CI]	CG D [CI]	-	-
Beyer et al., 2015	VISA-A	–14 ± 2.5 [–18.8, –8.8]	–22 ± 2.7 [–26.9, –16.4]	–27 ± 4.5 [–35.6, –18.0]	–34 ± 3.9 [–41.8, –26.5]	-	p = 0.62
	VAS (heel)	7 ± 3.9 [–0.8, 14.5]	22 ± 5.5 [10.8, 32.3]	13 ± 5.9 [1.3, 24.3]	24 6 5.7 [12.7, 35.0]	-	p = 0.77
	VAS (running)	29 ± 5.1 [18.9, 38.8]	37 ± 6.7 [23.4, 49.8]	38 ± 6.2 [25.6, 49.9]	49 ± 7.0 [35.5, 62.8]	-	p = 0.71
	A-P, mm	0 ± 0.1 [0, 0.5]	0.6 ± 0.2 [0.2, 0.9]	1.0 ± 0.3 [0.5, 1.5]	1.7 ± 0.3 [1.1, 2.4]	-	-
	Doppler, %	0 ± 0.4 [–0.9, 0.8]	2.3 ± 0.8 [0.8, 3.7]	4.0 ± 0.9 [2.7, 6.1]	1.1 ± 0.4 [0.2, 1.9]	-	-
Yu et al., 2012	YOU GO	-3.56 ± 0.60	-2.46 ± 0.66	-	-	-	-
	PTKE, Nm	37.47 ± 20.13	30.35 ± 36.43	-	-	p =0.636	-
	PTKF, Nm	10.48 ± 13.10	7.68 ± 18.83	-	-	p =0.735	-
	PTAP, Nm	9.47 ± 11.04	7.97 ± 10.94	-	-	p =0.789	-
	PTAD, Nm	7.68 ± 6.47	7.11 ± 12.79	-	-	p =0.911	-
	BAG, Nm	4.40 ± 20.13	-0.77 ± 28.28	-	-	p =0.680	-
	EKF, Nm	3.91 ± 18.40	0.61 ± 30.08	-	-	p =0.795	-
	EAP, Nm	13.56 ± 9.44	6.50 ± 10.46	-	-	p =0.178	-
	EAD, Nm	17.23 ± 5.47	8.70 8.01a	-	-	p =0.026	-
	Total balance index	-28.38 ± 6.73	-6.50 ± 11.61	-	-	X^2/P = 0.000	-
	AP balance index	-30.50 ± 16.26	-10.00 ± 23.54	-	-	X^2/P = 0.062	-
	ML balance index	-21.13 ± 10.93	-12.25 ± 9.13	-	-	X^2/P = 0.100	-
	Dexterity	23.88 ± 2.35	20.38 ± 4.43	-	-	X^2/P =0.058	-
	Agility	14.88 ± 2.69	9.75 ± 2.31	-	-	X^2/P = 0.001	-

Abbreviations: E.G, Experimental Group; C.G, Control Group, VISA-A, Victorian Institute of Sports Assessment–Achilles; VAS, visual analog scale; AP, anterior-posterior, PTKE, peak torque of knee extension; PTKF, peak torque of knee flexion; PTAP, peak torque of ankle plantar flexion; PTAD, peak torque of ankle, dorsiflexion; EKE, endurance of knee extension; EKF, endurance of knee flexion; EAP, endurance of ankle plantar flexion; EAD, endurance of ankle dorsiflexion.
